# The workload change and depression among emergency medical staff after the open policy during COVID-19: a cross-sectional survey in Shandong, China

**DOI:** 10.3389/fpubh.2023.1281787

**Published:** 2023-11-03

**Authors:** Baobao Feng, Hongjun Bian, Ke Zhang, Chong Meng, Xianwei Gong, Xueqiang Ma, Chunhua Su, Mingxiang Zhou, Jiarui Xu, Wei Zhang, Xingguo Zhang, Yi Zhou, Deya Shang

**Affiliations:** ^1^Department of Emergency, Shandong Provincial Hospital Affiliated to Shandong First Medical University, Jinan, China; ^2^Department of Pharmacy, Shandong Provincial Hospital Affiliated to Shandong First Medical University, Jinan, China; ^3^Department of Emergency Internal Medicine, The Affiliated Hospital of Qingdao University, Qingdao, China; ^4^Department of Emergency, Qilu Hospital of Shandong University Dezhou Hospital, Dezhou, China; ^5^Department of Emergency, Shandong Public Health Clinical Center Affiliated to Shandong University, Jinan, China

**Keywords:** COVID-19, overload, emergency medical staff, PHQ-9, open policy

## Abstract

**Introduction:**

In the middle of December 2022, the Chinese government adjusted the lockdown policy on coronavirus disease 2019 (COVID-19), a large number of infected patients flooded into the emergency department. The emergency medical staff encountered significant working and mental stress while fighting the COVID-19 pandemic. We aimed to investigate the workload change, and the prevalence and associated factors for depression symptoms among emergency medical staff after the policy adjustment.

**Methods:**

We conducted a cross-sectional online survey of emergency medical staff who fought against COVID-19 in Shandong Province during January 16 to 31, 2023. The respondents’ sociodemographic and work information were collected, and they were asked to complete the 9-item Patient Health Questionnaire (PHQ-9) then. Univariate and multivariate logistic regression analyses were applied to identify the potential associated factors for major depression.

**Results:**

Nine hundred and sixteen emergency medical personnel from 108 hospitals responded to this survey. The respondents’ weekly working hours (53.65 ± 17.36 *vs* 49.68 ± 14.84) and monthly night shifts (7.25 ± 3.85 *vs* 6.80 ± 3.77) increased after the open policy. About 54.3% of the respondents scored more than 10 points on the PHQ-9 standardized test, which is associated with depressive symptoms. In univariate analysis, being doctors, living with family members aged ≤16 or ≥ 65 years old, COVID-19 infection and increased weekly working hours after the open policy were significantly associated with a PHQ-9 score ≥ 10 points. In the multivariate analysis, only increased weekly working hours showed significant association with scoring ≥10 points.

**Conclusion:**

Emergency medical staff’ workload had increased after the open policy announcement, which was strongly associated with a higher PHQ-9 scores, indicating a very high risk for major depression. Emergency medical staff working as doctors or with an intermediate title from grade-A tertiary hospitals had higher PHQ-9 scores, while COVID-19 infection and weekly working hours of 60 or more after the open policy were associated with higher PHQ-9 scores for those from grade-B tertiary hospitals. Hospital administrators should reinforce the importance of targeted emergency medical staff support during future outbreaks.

## Introduction

The coronavirus disease 2019 (COVID-19) was first reported in Wuhan, China, in December 2019. It spread across the world and caused hundreds of millions infections since then. According to the World Health Organization (WHO), there have been 768,983,095 confirmed cases of COVID-19, including 6,953,743 deaths, by August 2nd, 2023 (1). In China, there have been 99,300,040 confirmed cases (including 121,563 deaths) by August 2nd, 2023 (1). Social distancing was recommended as one control option by the WHO to reduce the possibility of infection (2). However, hospital medical staff, especially those from emergency department who fight against COVID-19 on the front-lines, are unable to follow guidance on social distancing. In the middle of December 2022, the Chinese government adjusted the lockdown policy on COVID-19, a large number of infected patients flooded into the emergency department. Emergency medical staff were exposed to a high risk of coronavirus infection while suffering great working and mental stress. Previous infectious disease pandemics such as severe acute respiratory syndrome (SARS) and Middle East respiratory syndrome (MERS) have been reported to have a negative effect on people’s mental health, including medical staff (3, 4). COVID-19 pandemics have increased the prevalence of anxiety and depression among hospital medical staff (5–10). However, research exploring the mental health problems of front-line emergency medical staff after the open policy is limited. The aim of this research was to investigate the workload change, and the prevalence and associated factors for depressive symptoms among emergency medical staff during the policy adjustment period.

## Methods

### Study design and participants

This was a cross-sectional survey conducted in Shandong Province, China, between January 16 and January 31, 2023. The study was approved by the ethics committee of Shandong Provincial Hospital Affiliated to Shandong First Medical University (number SWYX:NO.2023–118), and all participants provided informed consent. Emergency medical staff (including doctors, nurses and pre-hospital emergency personnel) aged above 18 years who fought against COVID-19 between December 1, 2022 and January 15, 2023 were invited to participate. Those who were diagnosed with any mental illness previously or taking any anti-psychotic medications were excluded.

### Survey instrument

An online questionnaire system (Wenjuanxing, Changsha Ranxing Information Technology Co., LTD, Changsha, China) was used as the platform for distributing our survey tool. We sent the survey out through emergency medicine groups on the WeChat messaging platform (Tencent Corporation, Shenzhen, China). Then the Wenjuanxing system was able to collect the survey data electronically. Our survey instrument began with collecting participants’ general characteristics, including sex, age, occupation, marital status, and whether they live with family members younger than 16 years or older than 65 years. Then the respondents were queried about work details including grade of employing hospital, professional qualifications, working years, vaccination and COVID-19 infection status. We then collected their weekly working hours and monthly night shifts information before and after the open policy. At last, we used the Patient Health Questionnaire-9 (PHQ-9) standardized questionnaire to ascertain the mental state of surveyed emergency medical staff, which was a self-rated version of the Primary Care Evaluation of Mental Disorders (PRIME-MD) patient questionnaire for depression (11, 12). The PHQ-9 is scored 0–27, with the cutoff score for major depression symptoms in prior studies was set at 10 (13, 14). We used the standard score of ≥10 as the critical value to divide those participants with or without depression in this study.

### Statistical analysis

Continuous variables were presented as mean and standard deviation for normally distributed data or median and inter-quartile ranges for skewed data, while categorical variables were presented as frequency and percentages. Participants’ characteristics were compared according to PHQ-9 scoring ≥10 or not, using Student’s t-tests for quantitative variables and Chi-square tests for categorical variables. Univariate logistic regression analysis was performed to evaluate the relationships between variables and depressive symptoms. Variables associated with PHQ-9 ≥ 10 in univariate analysis (*p* < 0.10) were included in the multivariate model. *p* < 0.05 was considered statistically significant. All analyses were conducted using SPSS 20.0 software (IBM Inc., Armonk, NY, United States).

## Results

Nine hundred and sixty-one respondents completed the questionnaires through the online survey system, of which 916 (95.32%) were valid. Respondents are emergency medical staff from 108 hospitals located in all 16 cities of Shandong Province. The average PHQ-9 score for all included medical staff was 11.18 ± 6.50, and 497 participants (54.26%) had a PHQ-9 score ≥ 10. The prevalence of major depression symptoms was high with a PHQ-9 score distribution of 10–14 (26.75%), 15–19 (15.17%), and 20–27 (12.34%).

### Participants’ characteristics

Participants’ characteristics are shown in Table 1. Among these respondents, 46.72% were male and most (72.38%) were ≤ 40 years old. Doctors and nurses accounted for 43.56 and 52.18%, respectively. About two thirds (66.92%) of the participants lived with children ≤16 years old, while 47.05% of them lived with elders ≥65 years old. Most of the participants (77.84%) worked in tertiary hospitals. Junior and intermediate level personnel accounted for 40.39 and 43.23% respectively, while associate senior and senior level personnel accounted for 13.65 and 2.73%, respectively.

**Table 1:** Participants’ characteristics and univariate analysis results between the PHQ-9 < 10 and PHQ-9 ≥ 10 groups.

Characteristics	Overall (*n* = 916)	Overall PHQ-9 score	PHQ-9 < 10 (*n* = 419)	PHQ-9 ≥ 10 (*n* = 497)	*p* value
Sex					0.368
Male	428 (46.72%)	11(7, 16)	189 (44.16%)	239 (55.84%)	
Female	488 (53.28%)	10(7, 14)	230 (47.13%)	258 (52.87%)	
Age, years					0.61
18–30	222 (24.24%)	10(6, 14)	108 (48.65%)	114 (51.35%)	
31–40	441 (48.14%)	10(7, 16)	199 (45.12%)	242 (54.88%)	
41–50	191 (20.85%)	11(6, 16)	82 (42.93%)	109 (57.07%)	
51–60	61 (6.66%)	10(5.5, 15.5)	29 (47.54%)	32 (52.46%)	
> 60	1 (0.11%)	8	1 (100%)	0 (0.00%)	
Occupation					0.017
Doctor	399 (43.56%)	11(7, 16)	164 (41.10%)	235 (58.90%)	
Nurse	478 (52.18%)	9(6, 14)	240 (50.21%)	238 (49.79%)	
Others	39 (4.26%)	14(8, 19)	15 (38.46%)	24 (61.54%)	
Marital status					0.375
Married	750 (81.88%)	11(7, 16)	329 (43.87%)	421 (56.13%)	
Single	153 (16.70%)	9(6, 14)	81 (52.94%)	72 (47.06%)	
Divorced or widowed	13 (1.42%)	7(5, 16)	9 (69.23%)	4 (30.77%)	
Living with children ≤16y					0.021
Yes	613 (66.92%)	11(7, 16)	264 (43.07%)	349 (56.93%)	
No	303 (33.08%)	9(6, 14)	155 (51.16%)	148 (48.84%)	
Living with elders ≥65y					0.011
Yes	431 (47.05%)	11(7, 17)	178 (41.30%)	253 (58.70%)	
No	485 (52.95%)	10(6, 14)	241 (49.69%)	244 (50.31%)	
Grade of employing hospital					0.625
Grade-A Tertiary	442 (48.25%)	10(7, 16)	195 (44.12%)	247 (55.88%)	
Grade-B Tertiary	271 (29.59%)	10(6, 15)	129 (47.60%)	142 (52.40%)	
Grade-A Secondary and others	203 (22.16%)	10(7, 16)	95 (46.80%)	108 (53.20%)	
Professional qualifications					0.33
Junior	370 (40.39%)	10(7, 14)	180 (48.65%)	190 (51.35%)	
Intermediate	396 (43.23%)	11(8, 16)	168 (42.42%)	228 (57.58%)	
Associate senior	125 (13.65%)	10(6, 15)	58 (46.40%)	67 (53.60%)	
Senior	25 (2.73%)	9(4, 15.5)	13 (52.00%)	12 (48.00%)	
Working years					0.721
0–5	198 (21.62%)	10(6, 14)	94 (47.47%)	104 (52.53%)	
6–10	225 (24.56%)	10(7, 15)	109 (48.44%)	116 (51.56%)	
11–15	215 (23.47%)	10(7, 15)	96 (44.65%)	119 (55.35%)	
16–20	119 (12.99%)	11(6, 16)	49 (41.18%)	70 (58.82%)	
> 20	159 (17.36%)	11(6, 16)	71 (44.65%)	88 (55.35%)	
Total vaccination					0.596
Yes	889 (97.05%)	10(7, 15)	408 (45.89%)	481 (54.11%)	
No	27 (2.95%)	10(8, 17)	11 (40.74%)	16 (59.26%)	
COVID-19 infection					0.006
Yes	850 (92.79%)	10(7, 15)	378 (44.47%)	472 (55.53%)	
No	66 (7.21%)	8(3, 13)	41 (62.12%)	25 (37.88%)	
Weekly working hours before open policy					0.757
≤ 40	321 (35.04%)	11(7, 15)	151 (47.04%)	170 (52.96%)	
40–60	467 (50.98%)	10(7, 15)	208 (44.54%)	259 (55.46%)	
> 60	128 (13.97%)	10(7, 15)	60 (46.88%)	68 (53.12%)	
Monthly night shifts before open policy					0.35
≤ 5	267 (29.15%)	11(7, 16)	132 (49.44%)	135 (50.56%)	
6–10	525 (57.31%)	10(7, 15)	233 (44.38%)	292 (55.62%)	
> 10	124 (13.54%)	9(5, 13.75)	54 (43.55%)	70 (56.45%)	
Weekly working hours after open policy					0.009
≤ 40	255 (27.84%)	10(7, 15)	130 (50.98%)	125 (49.02%)	
40–60	441 (48.14%)	10(7, 15)	207 (46.94%)	234 (53.06%)	
> 60	220 (24.02%)	10(7, 16)	82 (37.27%)	138 (62.73%)	
Monthly night shifts after open policy					0.159
≤ 5	241 (26.31%)	11(8, 16)	121 (50.21%)	120 (49.79%)	
6–10	529 (57.75%)	10(7, 15.5)	239 (45.18%)	290 (54.82%)	
> 10	146 (15.94%)	9(5, 13.25)	59 (40.41%)	87 (59.59%)	
Overall	916 (100%)	10(7, 15)	419 (45.74%)	497 (54.26%)	

### Workload before and after the open policy

The workload change during the open policy are shown in Table 2. Before the open policy, 321 (35.04%), 467 (50.98%) and 128 (13.97%) participants’ weekly working hours were ≤ 40, 40–60, and > 60 h, respectively. Of all the included participants, 267 (29.15%) had a monthly night shifts of ≤5, while 525 (57.31%) of them had a monthly night shifts of 6–10. After the open policy announcement, 441 (48.14%) participants’ weekly working hours were 40–60 h, while 220 (24.02%) participants’ were > 60 h. And 241 (26.31%) participants had a monthly night shifts of ≤5, while 529 (57.75%) of them had a monthly night shifts of 6–10. Overall, the participants’ weekly working hours increased after the open policy (53.65 ± 17.36 *vs* 49.68 ± 14.84, *p* < 0.001), which was the same for monthly night shifts(7.25 ± 3.85 *vs* 6.80 ± 3.77, *p* < 0.001). For different subgroups (sex, age, employing hospital, etc), the results were similar.

**Table 2:** The workload change before and after the open policy.

Variables	Weekly working hours			Monthly night shifts		
	Before	After	*p* value	Before	After	*p* value
Sex						
Male	52.19 ± 15.33	58.17 ± 17.56	< 0.001	6.95 ± 3.85	7.67 ± 3.85	< 0.001
Female	47.47 ± 14.04	49.67 ± 16.18	< 0.001	6.67 ± 3.71	6.88 ± 3.82	0.015
Age, years						
18–30	48.73 ± 14.40	50.50 ± 16.84	0.003	7.74 ± 3.37	8.24 ± 5.54	0.118
31–40	49.16 ± 14.63	52.09 ± 16.31	< 0.001	7.37 ± 3.36	7.91 ± 3.78	< 0.001
41–50	52.65 ± 15.41	58.32 ± 17.35	< 0.001	5.59 ± 4.14	6.27 ± 4.08	< 0.001
> 50	47.60 ± 15.19	55.79 ± 22.87	< 0.001	3.11 ± 3.64	3.97 ± 4.24	0.007
Grade of employing hospital						
Grade-A Tertiary	46.45 ± 12.54	49.57 ± 15.13	< 0.001	6.26 ± 3.26	6.71 ± 3.43	< 0.001
Grade-B Tertiary	52.75 ± 17.21	58.08 ± 18.15	< 0.001	7.46 ± 4.27	8.07 ± 4.16	< 0.001
Grade-A Secondary and others	52.60 ± 14.65	56.59 ± 18.79	< 0.001	7.12 ± 3.96	7.32 ± 4.10	0.259
Occupation						
Doctor	51.90 ± 14.08	58.76 ± 17.83	< 0.001	6.11 ± 3.66	7.00 ± 3.74	< 0.001
Nurse	46.52 ± 13.52	48.41 ± 14.82	< 0.001	7.06 ± 3.65	7.18 ± 3.79	0.116
Others	65.64 ± 22.19	65.51 ± 20.10	0.959	10.72 ± 3.71	10.64 ± 4.11	0.653
Professional qualifications						
Junior	50.23 ± 16.91	52.43 ± 18.32	< 0.001	7.98 ± 3.50	8.12 ± 3.66	0.125
Intermediate	49.42 ± 12.42	53.56 ± 14.42	< 0.001	6.99 ± 3.29	7.55 ± 3.45	< 0.001
Associate senior	48.54 ± 15.57	56.26 ± 21.52	< 0.001	3.60 ± 3.60	4.53 ± 3.82	< 0.001
Senior	51.28 ± 13.74	59.96 ± 20.31	0.002	2.44 ± 3.82	3.08 ± 4.15	0.218
Working years						
0–5	49.48 ± 15.54	51.37 ± 18.01	0.005	7.57 ± 3.42	7.69 ± 3.69	0.422
6–10	49.46 ± 14.49	52.32 ± 14.43	< 0.001	7.68 ± 3.39	8.02 ± 3.40	0.001
11–15	48.93 ± 15.03	53.60 ± 17.82	< 0.001	7.27 ± 3.34	8.03 ± 4.14	0.001
16–20	50.29 ± 13.04	54.76 ± 15.97	< 0.001	6.38 ± 3.91	6.99 ± 3.70	< 0.001
> 20	50.78 ± 15.51	57.58 ± 19.98	< 0.001	4.31 ± 4.07	4.96 ± 4.29	< 0.001
Overall	49.68 ± 14.84	53.65 ± 17.36	< 0.001	6.80 ± 3.77	7.25 ± 3.85	< 0.001

Male staff took more workload than female (eg. weekly working hours after the open policy among men vs. women: 58.17 ± 17.56 vs. 49.67 ± 16.18). Doctors had longer weekly working hours than nurses before and after the policy adjustment (eg. weekly working hours after the open policy among doctors vs. nurses: 58.76 ± 17.83 vs. 48.41 ± 14.82). Though doctors took less night shifts than nurses before the open policy (6.11 ± 3.66 vs. 7.06 ± 3.65), their night shifts increased to the same level as nurses after the open policy (7.00 ± 3.74 vs. 7.18 ± 3.79). The weekly working hours and monthly night shifts of grade A tertiary hospitals’ emergency medical staff were less than the other two hospital groups (both before and after the open policy).

### COVID-19 infection

**Figure 1:**
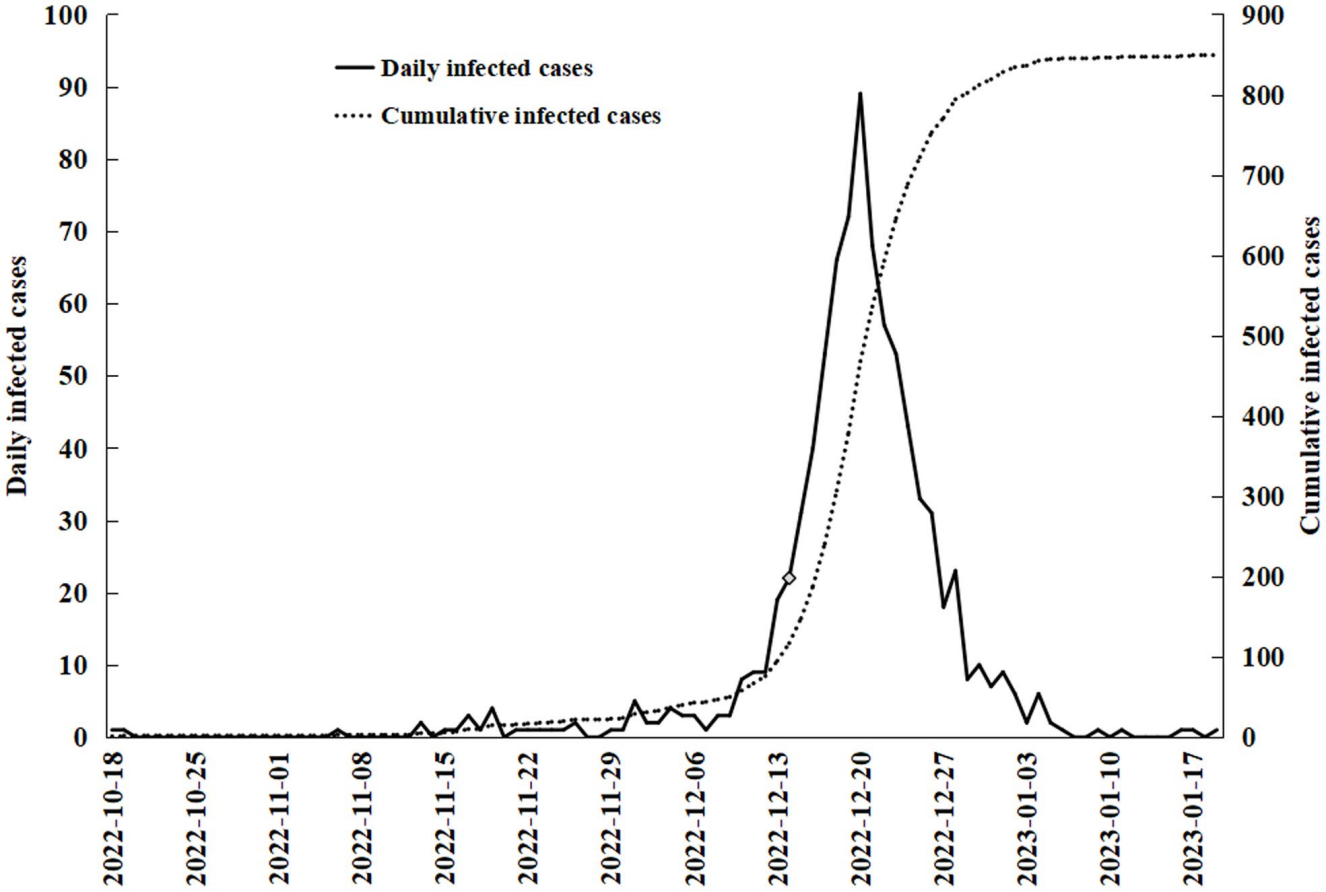
The COVID-19 infection of emergency medical staff.

Almost all (97.05%) of the medical staff surveyed completed the full course of vaccination, but most of them (92.79%) were still infected with novel coronavirus. Before the government announced the open policy, infections with COVID-19 among health workers were at a low level. After the announcement of the open policy, a large number of infected patients flooded into the emergency department, and infections among emergency medical staff were skyrocketing (Figure 1).

Before December 14th, 2022, there were 95(10.37%) respondents who had been infected with COVID-19, while 666(72.71%) had a contact history of confirmed COVID-19 patients. Since then, the number of daily infections among emergency medical staff increased significantly, peaking at 89 on December 20th, 2022 (Figure 1). By January 19th, 2023, 887(96.83%) respondents reported a contact history of confirmed COVID-19 patients, 850(95.83%) of whom were infected with COVID-19 (Table 3). Most COVID-19 infections among emergency medical staff were confirmed by positive nucleic acid (54.47%) or antigen (26.12%). One hundred and fifteen participants took a chest computed tomography, among whom 40(34.78%) had image findings of COVID-19. After contracting COVID-19, most participants had to continue working due to the desperately shortage of medical personnel. There were 378(44.47%) respondents who left the work position to have a rest for 1–3 days, while 106(12.47%) respondents did not rest at all. Only 95(11.18%) participants returned to work after they had recovered.

**Table 3:** COVID-19 infection of emergency medical staff.

	Number (%)
Contact history of confirmed COVID-19	
Before December 14th, 2022	666 (72.71)
After December 14th, 2022	887 (96.83)
COVID-19 infection	
Non-infected	66 (7.21)
Infected before December 14th, 2022	95 (10.37)
Infected after December 14th, 2022	755 (82.42)
Diagnosis^a^	
Positive nucleic acid	463 (54.47)
Positive antigen	222 (26.12)
Clinical diagnosis	165 (19.41)
Chest computed tomography^a^	
Non-available	735 (86.47)
Normal	75 (8.82)
Novel coronavirus pneumonia	40 (4.71)
Rest time after contracting COVID-19 (days)^a^	
0	106 (12.47)
1–3	378 (44.47)
4–7	331 (38.94)
>7	35 (4.12)
Healed when returned to work^a^	
Yes	95 (11.18)
No	755 (88.82)

a *N* = 850.

### Factors associated with depressive symptoms among emergency medical staff

Univariate analysis of factors associated with major depression symptoms among emergency medical staff are shown in Table 1. Being doctors, living with family members younger than 16 years or older than 65 years, COVID-19 infection and increased weekly working hours after the open policy showed statistical significance. There was no significant difference in the PHQ-9 scores divided by prevalence according to sex, age, marital status, grade of employing hospital, professional qualifications, working years and monthly night shifts. Multivariate logistic regression analysis showed that a PHQ-9 score ≥ 10 was significantly associated with increased weekly working hours after the open policy. Results of the multivariate logistic regression analysis are shown in Table 4.

**Table 4:** Factors associated with depression symptoms by multivariate logistic regression analysis.

Variables	Odds ratio	95% Confidence interval	*p* value
Weekly working hours after open policy			0.009
≤ 40	1	–	–
40–60	1.750	1.212–2.527	0.003
> 60	1.489	1.069–2.073	0.019

### Comparisons among hospitals of different grades

The results of comparisons among different hospital groups are shown in Table 5. For emergency medical staff working in grade A tertiary hospitals, being doctors, living with family member ≥65y, an intermediate professional qualifications, increased weekly working hours and monthly night shifts after the policy adjustment were associated with a higher PHQ-9 score. For those working in grade B tertiary hospitals, being unvaccinated, COVID-19 infection and increased weekly working hours after the open policy showed a strong relationship with PHQ-9 ≥ 10. No factors were found to be associated with a higher PHQ-9 score for those working in grade A secondary and other hospitals. When individual factors were compared among hospitals, those from grade A tertiary hospitals had a lower proportion of junior professional qualifications and vaccination than others. The weekly working hours and monthly night shifts of grade A tertiary hospitals’ medical staff were less than others (both before and after the open policy). Though no statistical significance among different groups was observed, medical staff with weekly working hours of 40 to 60 h and monthly night shifts of >10 from grade A tertiary hospitals tended to have a higher PHQ-9 score.

**Table 5:** Intergroup comparisons of hospital grades.

Characteristics	Grade-A Tertiary (*n* = 442)				Grade-B Tertiary (*n* = 271)				Grade-A Secondary and others (*n* = 203)					*p* value
	*n*	PHQ-9		p^*^ value	*n*	PHQ-9		p^*^ value	*n*	PHQ-9		p^*^ value	p^**^ value	
		< 10	≥ 10			< 10	≥ 10			< 10	≥ 10			
Sex				0.133				0.672				0.78		0.456
Male	199	80	119		135	66	69		94	43	51		0.275	
Female	243	115	128		136	63	73		109	52	57		0.973	
Age, years				0.471				0.934				0.836		0.124
18–30	106	50	56		54	26	28		62	32	30		0.940	
31–40	224	96	128		135	66	69		82	37	45		0.539	
41–50	81	32	49		65	30	35		45	20	25		0.703	
51–60	30	16	14		17	7	10		14	6	8		0.669	
> 60	1	1	0		0	0	0		0	0	0		NA	
Occupation				0.026				0.897				0.163		0.075
Doctor	176	66	110		130	60	70		93	38	55		0.314	
Nurse	245	122	123		127	62	65		106	56	50		0.816	
Others	21	7	14		14	7	7		4	1	3		0.515	
Marital status				0.053				0.155				0.513		0.409
Married	372	155	217		219	101	118		159	73	86		0.484	
Single	64	37	27		49	25	24		40	19	21		0.561	
Divorced or widowed	6	3	3		3	3	0		4	3	1		0.296	
Living with children ≤16y				0.053				0.065				0.992		0.204
Yes	298	122	176		189	83	106		126	59	67		0.514	
No	144	73	71		82	46	36		77	36	41		0.494	
Living with elders ≥65y				0.047				0.263				0.171		0.054
Yes	191	74	117		142	63	79		98	41	57		0.584	
No	251	121	130		129	66	63		105	54	51		0.795	
Professional qualifications				0.023				0.975				0.324		0.035
Junior	156	75	81		118	58	60		96	47	49		0.982	
Intermediate	207	77	130		116	54	62		73	37	36		0.076	
Associate senior	64	33	31		30	15	15		31	10	21		0.189	
Senior	15	10	5		7	2	5		3	1	2		0.518	
Working years				0.733				0.460				0.940		0.274
0–5	91	44	47		51	22	29		56	28	28		0.757	
6–10	115	51	64		72	40	32		38	18	20		0.325	
11–15	107	43	64		62	31	31		46	22	24		0.413	
16–20	55	22	33		40	16	24		24	11	13		0.874	
> 20	74	35	39		46	20	26		39	16	23		0.801	
Total vaccination				0.316				0.015						0.006
Yes	422	184	238		264	129	135		203	95	108		0.387	
No	20	11	9		7	0	7		0	0	0		0.022	
COVID-19 infection				0.287				0.030				0.067		0.874
Yes	410	178	232		253	116	137		187	84	103		0.821	
No	32	17	15		18	13	5		16	11	5		0.336	
Weekly working hours before open policy				0.099				0.471				0.567		< 0.001
≤ 40	196	96	100		63	29	34		62	26	36		0.616	
40–60	209	81	128		161	81	80		97	46	51		0.070	
> 60	37	18	19		47	19	28		44	23	21		0.510	
Monthly night shifts before open policy				0.065				0.901				0.535		< 0.001
≤ 5	139	68	71		71	34	37		57	30	27		0.854	
6–10	275	120	155		142	66	76		108	47	61		0.841	
> 10	28	7	21		58	29	29		38	18	20		0.077	
Weekly working hours after open policy				0.045				0.007				0.950		< 0.001
≤ 40	163	83	80		42	23	19		50	24	26		0.811	
40–60	214	90	124		136	74	62		91	43	48		0.078	
> 60	65	22	43		93	32	61		62	28	34		0.316	
Monthly night shifts after open policy				0.028				0.960				0.569		< 0.001
≤ 5	124	62	62		58	28	30		59	31	28		0.897	
6–10	279	123	156		150	72	78		100	44	56		0.714	
> 10	39	10	29		63	29	34		44	20	24		0.090	

^*^Comparison between the intra-hospital group. ^**^Comparison between the different hospital groups for individual parameters. NA, not applicable.

Multivariate logistic regression analysis showed that nurses and those with a senior title had a lower PHQ-9 score among emergency medical staff from grade A tertiary hospitals (Table 6). For those working in grade B tertiary hospitals, COVID-19 infection and increased weekly working hours (> 60) after the open policy were associated with higher PHQ-9 score (Table 7).

**Table 6:** Factors associated with depression symptoms by multivariate logistic regression analysis among emergency medical staff from grade-A tertiary hospitals.

Variables	Odds ratio	95% Confidence interval	*p* value
Occupation			0.004
Doctor	1		
Nurse	0.498	0.320–0.776	0.002
Others	1.066	0.393–2.894	0.900
Professional qualifications			0.005
Junior	1		
Intermediate	1.425	0.918–2.211	0.114
Associate senior	0.649	0.345–1.223	0.182
Senior	0.282	0.087–0.916	0.035

**Table 7:** Factors associated with depression symptoms by multivariate logistic regression analysis among emergency medical staff from grade-B tertiary hospitals.

Variables	Odds ratio	95% Confidence interval	*p* value
COVID-19 infection	3.008	1.019–8.881	0.046
Weekly working hours after open policy			0.005
≤ 40	1		
40–60	1.157	0.558–2.398	0.695
> 60	2.666	1.227–5.795	0.013

### Comparisons between doctors and nurses

Due to the small number of pre-hospital personnel and others, we made comparisons between doctors and nurses only (Table 8). Increased weekly working hours after the open policy were associated with PHQ-9 ≥ 10 for doctors, while marriage and COVID-19 infection showed a similar relationship with PHQ-9 ≥ 10 for nurses. Compared with nurses, doctors had a higher proportion of male sex, age of >40y, living with elders ≥65y, senior titles, long working years, and long weekly working hours. The unmarried rate and monthly night shifts before the open policy were higher for nurses. Interestingly, though nurses had more night shifts per month, their weekly working hours were lower than doctors (Table 2). The night shifts of doctors increased (7.00 ± 3.74 vs. 6.11 ± 3.66) after the open policy, while those of nurses did not change significantly (Table 2). When individual parameters were compared between doctors and nurses, doctors who were female, single, living with children ≤16y, working in grade A tertiary hospitals, having a senior title, infected with COVID-19, having longer weekly working hours and more night shifts were more prone to have a PHQ-9 ≥ 10 than nurses with the same conditions. Multivariate logistic regression analysis showed that nurses with associate senior titles had a lower PHQ-9 score than those with a junior or intermediate titles, though there was no statistical significance overall (Table 9). No factors were found to be associated with a higher PHQ-9 score among doctors in multivariate logistic regression analysis.

**Table 8:** Intergroup comparisons of doctors and nurses.

Characteristics	Doctor(n = 399)				Nurse(n = 478)					*p* value
	*n*	PHQ-9		p^*^ value	*n*	PHQ-9		p^*^ value	p^**^ value	
		< 10	≥ 10			< 10	≥ 10			
Sex				0.437				0.755		< 0.001
Male	311	131	180		87	45	42		0.111	
Female	88	33	55		391	195	196		0.036	
Age, years				0.683				0.643		< 0.001
18–30	47	17	30		170	89	81		0.069	
31–40	177	71	106		242	118	124		0.079	
41–50	124	53	71		57	27	30		0.56	
51–60	50	22	28		9	6	3		0.21	
> 60	1	1	0		0	0	0		NA	
Marital status				0.169				0.040		< 0.001
Married	362	150	212		356	168	188		0.121	
Single	35	12	23		113	65	48		0.016	
Divorced or widowed	2	2	0		9	7	2		> 0.99	
Living with children ≤16y				0.218				0.173		0.166
Yes	274	107	167		307	147	160		0.032	
No	125	57	68		171	93	78		0.135	
Living with elders ≥65y				0.079				0.115		< 0.001
Yes	218	81	137		186	85	101		0.082	
No	181	83	98		292	155	137		0.127	
Grade of employing hospital				0.314				0.816		0.077
Grade-A Tertiary	176	66	110		245	122	123		0.012	
Grade-B Tertiary	130	60	70		127	62	65		0.669	
Grade-A Secondary and others	93	38	55		106	56	50		0.091	
Professional qualifications				0.686				0.077		< 0.001
Junior	83	35	48		257	133	124		0.129	
Intermediate	190	75	115		198	91	107		0.197	
Associate senior	101	41	60		23	16	7		0.012	
Senior	25	13	12		0	0	0		NA	
Working years				0.817				0.534		< 0.001
0–5	59	21	38		130	69	61		0.026	
6–10	76	34	42		133	67	66		0.433	
11–15	93	36	57		115	58	57		0.091	
16–20	70	30	40		49	19	30		0.656	
> 20	101	43	58		51	27	24		0.226	
Total vaccination				0.097				0.986		0.126
Yes	391	163	228		460	231	229		0.013	
No	8	1	7		18	9	9		0.070	
COVID-19 infection				0.110				0.043		0.647
Yes	370	148	222		447	219	228		0.010	
No	29	16	13		31	21	10		0.317	
Weekly working hours before open policy				0.582				0.206		< 0.001
≤ 40	79	35	44		236	114	122		0.538	
40–60	261	108	153		192	95	97		0.087	
> 60	59	21	38		50	31	19		0.006	
Monthly night shifts before open policy				0.171				0.714		< 0.001
≤ 5	140	66	74		125	65	60		0.402	
6–10	229	88	141		273	138	135		0.038	
> 10	30	10	20		80	37	43		0.223	
Weekly working hours after open policy				0.012				0.713		< 0.001
≤ 40	53	26	27		197	102	95		0.725	
40–60	208	95	113		218	105	113		0.606	
> 60	138	43	95		63	33	30		0.004	
Monthly night shifts after open policy				0.248				0.314		0.129
≤ 5	110	52	58		127	67	60		0.400	
6–10	240	95	145		269	138	131		0.008	
> 10	49	17	32		82	35	47		0.366	

^*^Comparison between the intra-career group. ^**^Comparison between the doctor and nurse group for individual parameters. NA, not applicable.

**Table 9:** Factors associated with depression symptoms by multivariate logistic regression analysis among nurses.

Variables	Odds ratio	95% Confidence interval	*p* value
Marital status			0.069
Married	1		
Single	0.259	0.052–1.284	0.098
Divorced or widowed	0.391	0.075–2.036	0.265
Professional qualifications			0.118
Junior	1		
Intermediate	0.402	0.156–1.039	0.060
Associate senior	0.371	0.145–0.950	0.039
COVID-19 infection	2.091	0.953–4.587	0.066

## Discussion

This was a multi-center, cross-sectional study on the influence of policy adjustment on emergency medical staff, aiming to find the workload change, and the prevalence and risk factors for depression after the open policy during the COVID-19 pandemic in Shandong, China. The results showed that more than half of the surveyed respondents had a PHQ-9 score ≥ 10, which were consist with previous researches (9, 15). In our study, medical profession, living with juvenile or aged family members, COVID-19 infection and increased weekly working hours were associated with an increased PHQ-9 score in univariate analysis. However, only increased weekly working hours after the open policy showed a strong relationship with a PHQ-9 score ≥ 10 when multivariate analysis was carried out.

Previous study showed that the average working hours of all Chinese emergency medical staff were relatively long, with an average 12 h-long shift and 50 h of weekly working hours, which was consist with our survey results (9). Interestingly, the average working hours per week before COVID-19 pandemic were more than the average hours during COVID-19 in that study (8). The decreased working hours during COVID-19 pandemic might be explained by medical personnel support from other departments, national compulsory isolation policy or decreased emergency visits for other diseases other than fever (9, 16, 17). However, both weekly working hours and monthly night shifts of the respondents from our study had increased after the open policy announcement. There were several reasons for this. First, the emergency visits soared in a short time due to the rapid increasing infection. Second, since the lockdown policy had been lifted, patients with diseases other than fever came to emergency department seeking for care. Third, the emergency medical staff themselves were infected with COVID-19, some of whom had to leave their work position for several days due to poor physical conditions and the remaining ones shouldered their workloads. And increased workload was associated with higher rates of depression and other mental health outcomes, which had been verified in previous studies (18, 19).

Depression was one of the most common mental health problems among medial staff during COVID-19 pandemic and the reported prevalence varied from 13.4 to 53.9% in different researches (6, 7, 9, 10, 15, 20). There were different factors reported to be associated with depression, such as age, sex, marriage status, work position, etc. These factors might had a relationship with depression in a study, while not in another. However, front-line medical staff who participated in direct diagnosis, treatment and care of COVID-19 patients were reported to have a higher risk of depression in extensive literature (7, 9, 10, 15, 20). Emergency departments were the first presentation areas of patients before and during the COVID-19 pandemic period. Emergency medical staff working on the frontline during the pandemic experience more mental health problems than those working in other positions (4, 15, 21). Concur with our study, depression were detected in more than half of the participants in previous studies (4, 21).

Female, nurse and intermediate technical title were associated with worse mental health outcomes including depression, anxiety, and distress in a previous study (15). However, 76.7% of the participants were women, and 60.8% were nurses, there might be selection bias in that study (15). In our study, male accounted for nearly half (46.72%) of the whole participants, and 43.56% were doctors. The results showed that there was no difference between male and female for scores of depression, which was consist with a meta-analysis published in 2020 (20). Contrast with previous studies, doctors had higher PHQ-9 scores than nurses in our study. A meta-analysis of 26 studies investigating 31,447 doctors demonstrated that the prevalence of depression was 20.5% with the point prevalence ranged from 6.1 to 73.4% (22). Doctors, especially those working in grade A tertiary hospitals, had higher PHQ-9 scores than nurses in our study cohort. For front-line healthcare workers caring for COVID-19 patients, the prevalence of depression in doctors was much higher than nurses (40.4% vs. 28%), which was reported by a meta-analysis (5). As for our study cohort, the workload of doctors increased more significantly than that of nurses during the open policy, making them more stressed. They might experience poor job satisfaction, which was associated with higher prevalence of mental health problems (anxiety, depression and secondary traumatic stress) (23). For doctors from emergency department, the prevalence might be higher, since they need to make quick decisions on the diagnosis and treatment strategy when critically ill patients arrived in the emergency room, which make their occupational stress higher and result in psychological consequences such as depression and anxiety (24, 25). In our study cohort, emergency medical staff with associate senior (for nurses) or senior (for doctors) titles had a lower PHQ-9 score than those with an intermediate title. This could be explained by the fact that those with lower professional titles were more likely to work on the front-line.

Hospitals were the most common exposure sites, and medical staff have a higher risk for occupational COVID-19 compared with the general workforce (26). Researchers conducted an online survey on 1766 front-line nurses working in hospitals located in Shenzhen, China, after the open policy announcement. About 90.83% of the participants were infected with COVID-19, and 33.64% of them had to work while infected with COVID-19 (27). The overall prevalence of depressive symptoms was 69.20% (27). However, only 76 (4.3%) of the participants worked in the emergency departments (27). In our cohort, only 10.37% of the overall participants were infected before the adjustment of anti-epidemic policy at December 14th, 2022. Another 755 (82.42%) participants reported COVID-19 infection in the following month. Most of them (88.82%) had to return to work before getting healed due to the shortage of medical personnel.

A study surveying 1,103 emergency nurses showed that working in tertiary hospitals were significantly associated with depression (28). In another study, medical staffs in secondary hospitals reported higher scores for depression than those in tertiary hospitals (15). Despite of differences between studies, front-line medical staff from tertiary and secondary hospitals reported similar high PHQ-9 scores (15, 28), which was consist with our research. Emergency medical staff working in grade A tertiary hospitals undertook less weekly working hours and monthly night shifts than those working in the other two hospital groups, which could be explained by the concentration of medical resources. The higher proportion of medical personnel with intermediate titles in grade A tertiary hospitals also reflects the imbalance of medical resources. However, they did not have a lower PHQ-9 score, because they need to handle more patients with more complex and critical situations than those working in other hospitals.

Previous study has demonstrated that the ongoing stress have negative effects on medical staffs’ psychological well-being, especially when they face a great threat of public health emergencies (29). A longitudinal study conducted by Filippo Rapisarda and colleagues showed that psychological distress (a combination of severe post-traumatic, depressive and anxiety symptoms) tended to resolve within a few weeks, though it was present in 40% of healthcare workers (30). A Canadian study demonstrated similar result during one-year observation (31). Resilience and social support were predominant protective factors against depression over time (31). So health administrators should provide interventions to improve working conditions and reduce occupational stress of medical staffs to reduce or prevent prevalence of depression and other mental health problems.

Our study has some strengths. First, this study demonstrated the heavy workload and high prevalence of depression among emergency medical staff from China after the announcement of open policy, which highlighted the importance of targeted emergency medical staff support during future outbreaks. Second, the survey was conducted in a populous province. The population of Shandong is more than 100 million (about 7.19% of the total Chinese population), and the number of physicians accounts for 7.86% of the total number of physicians in China. Therefore, the research conducted in Shandong can be representative of China to a certain extent. Third, our study cohort included a higher proportion of male and doctors, which is less likely to be biased than previous studies that focused on female and nurses.

Our study has several limitations. First, the cross-sectional design only assessed individual status at the time the data were collected, which was not suitable for detecting intra-individual change across time and address causal associations. Second, there might be self-report bias since the participants completed the questionnaires online. Third, we only assessed depression in this study, other mental problems such as anxiety, insomnia and post-traumatic stress disorder were not included in the questionnaire. Too many questions included in the questionnaire might reduce the motivation of the respondents, since the survey was carried out during the peak of the epidemic after the open policy announcement. Fourth, the PHQ-9 scale is not accurate enough to make a definite diagnosis of depression. Scores above the threshold suggest a detailed psychological assessment. The prevalence estimates of common mental disorders such as anxiety and depression in medical staff were considerably lower when assessed using diagnostic interviews compared with screening tools (5). However, it was not realistic to conduct a diagnostic interview under the tense circumstances when the study was conducted. Fifth, the study included participants from Shandong Province only, and the results may not be applicable to other regions of China.

## Conclusion

Our study demonstrates that emergency medical staff’ workload had increased after the open policy announcement, which was strongly associated with higher PHQ-9 scores, indicating a very high risk for major depression. Emergency medical staff working as doctors or with an intermediate title from grade-A tertiary hospitals had higher PHQ-9 scores, while COVID-19 infection and weekly working hours of 60 or more after the open policy were associated with higher PHQ-9 scores for those from grade-B tertiary hospitals. Hospital administrators should reinforce the importance of targeted emergency medical staff support during future outbreaks.

## Data availability statement

The raw data supporting the conclusions of this article will be made available by the authors, without undue reservation.

## Ethics statement

The studies involving humans were approved by the ethics committee of Shandong Provincial Hospital Affiliated to Shandong First Medical University. The studies were conducted in accordance with the local legislation and institutional requirements. The ethics committee/institutional review board waived the requirement of written informed consent for participation from the participants or the participants’ legal guardians/next of kin because The study was conducted online during the peak of the epidemic after the open policy announcement. The participants were asked to provide oral informed consent at the beginning of the online questionnaire. If they disagreed, they didn’t need to fill out a questionnaire.

## Author contributions

BF: Conceptualization, Data curation, Formal analysis, Investigation, Methodology, Project administration, Resources, Supervision, Validation, Writing – original draft, Writing – review & editing. HB: Data curation, Formal analysis, Supervision, Validation, Writing – review & editing. KZ: Data curation, Formal analysis, Supervision, Validation, Writing – review & editing. CM: Data curation, Formal analysis, Investigation, Writing – review & editing. XG: Data curation, Formal analysis, Investigation, Methodology, Writing – review & editing. XM: Investigation, Methodology, Validation, Writing – review & editing. CS: Data curation, Formal analysis, Investigation, Supervision, Writing – review & editing. MZ: Data curation, Formal analysis, Investigation, Validation, Writing – review & editing. JX: Data curation, Investigation, Methodology, Writing – review & editing. WZ: Data curation, Investigation, Methodology, Writing – review & editing. XZ: Data curation, Project administration, Supervision, Validation, Writing – review & editing. YZ: Conceptualization, Methodology, Project administration, Supervision, Writing – review & editing. DS: Conceptualization, Methodology, Project administration, Supervision, Writing – review & editing.
